# Small Burns Need Attention Too: Evaluating the 15% Burn Resuscitation Threshold in Adults

**DOI:** 10.1093/jbcr/iraf185

**Published:** 2025-09-29

**Authors:** Ashleigh Bull, Mala Sharma, Alexander Kurjatko, Sarah Wellsandt, Brooke Dwars, Colette Galet, Lucy Wibbenmeyer

**Affiliations:** Department of Surgery, University of Iowa, Iowa Health Care, Iowa City, IA 52246, USA; Department of Nursing, University of Iowa, Iowa Health Care, Iowa City, IA 52246, USA; Department of Surgery, University of Iowa, Iowa Health Care, Iowa City, IA 52246, USA; Department of Nursing, University of Iowa, Iowa Health Care, Iowa City, IA 52246, USA; Department of Surgery, University of Iowa, Iowa Health Care, Iowa City, IA 52246, USA; Department of Nursing, University of Iowa, Iowa Health Care, Iowa City, IA 52246, USA; Department of Surgery, University of Iowa, Iowa Health Care, Iowa City, IA 52246, USA; Department of Nursing, University of Iowa, Iowa Health Care, Iowa City, IA 52246, USA; Department of Surgery, University of Iowa, Iowa Health Care, Iowa City, IA 52246, USA; Department of Nursing, University of Iowa, Iowa Health Care, Iowa City, IA 52246, USA; Department of Surgery, University of Iowa, Iowa Health Care, Iowa City, IA 52246, USA; Department of Nursing, University of Iowa, Iowa Health Care, Iowa City, IA 52246, USA; Department of Surgery, University of Iowa, Iowa Health Care, Iowa City, IA 52246, USA; Department of Nursing, University of Iowa, Iowa Health Care, Iowa City, IA 52246, USA

**Keywords:** burn injury, resuscitation, over resuscitation, resuscitation protocols, vasopressors, albumin, colloid

## Abstract

The American Burn Life Support course recommends fluid resuscitation of patients with TBSA ≥20% to prevent burn shock. Our center resuscitates patients with burns greater than 15% TBSA. Herein, we characterize that population. Patients with burns 15% to 19.9% TBSA admitted from January 1, 2019 to March 31, 2023 who received protocolized fluid resuscitation were included. Demographics, hospital course, and fluids received were reviewed. Fluid resuscitation was categorized as “below range” (Parkland formula [PF] < 3 mL/kg/%TBSA), “within range” (PF = 3-5 mL/kg/%TBSA or “above range” (PF > 5 mL/kg/%TBSA). Similarly, urine output (UOP) was expressed as “below range” (<30 mL/h), “within range” (31-50 mL/h) or “above range” (>50 mL/h). The resuscitation groups were compared. *P* < .05 was considered significant. Thirty-three patients received resuscitation via Brooke (9.1%), PF (63.6%), or other formula (27.3%). Most were male (81.8%) with a median TBSA of 17%; median age was 57 years. Almost 20% of patients required vasopressors during resuscitation. Fifteen patients were within the predicated range of PF, 15 were under, and 3 were over. There was no difference between the groups with respect to demographics, burn injury variables, or complications. Notably, the average creatinine and lactate 24 h postadmission were 0.9 mg/dL and 2 mg/dL, respectively. Half of the study patients received greater than maintenance; all were in either the within burn resuscitation range or above range groups. This retrospective study suggests that patients with smaller burns may benefit from resuscitation as 50% received more than maintenance. Resuscitation of smaller burns requires more study.

## INTRODUCTION

Following a burn injury, derangements in oncotic and hydrostatic pressures, a surge in inflammatory mediators, and a dysfunctional microvasculature can lead to burn shock without adequate resuscitation.^1^ In 1968, Baxter and Shires introduced the Parkland formula (PF), which has since become the cornerstone of fluid resuscitation in the initial treatment of burn pathology.^2^ Since then, the focus on burn resuscitation has been on which formula to use, the addition of colloid, and the endpoints to target.^3^^,^^4^ As a result of these interventions, the amount of resuscitation patients received over the ensuing years has been likened to a swinging pendulum, ebbing, and flowing based on these variables.^5^^,^^6^ Pruitt’s work, along with that of others, helped tighten the administration of resuscitation fluids.^4^^,^^5^ The focus of these studies has been almost entirely on burns ≥20% TBSA burned.

While there is a general agreement that patients with TBSA ≥20% warrant resuscitation, there is scant literature on resuscitation of burns <20% TBSA.^7^^,^^8^ Reflecting the lack of scientific study of smaller burns, a telephone survey of United Kingdom burn centers showed discordance regarding the TBSA trigger to initiate resuscitation.^9^ The majority of the respondents began resuscitation at 15% TBSA (71.4%) with less than 21.5% starting at the usual 20% TBSA trigger. In patients with TBSA <20%, individual patient characteristics may influence the need for fluids. While smaller burns have not been studied extensively, inhalation injury (INH), alcohol and illicit drug use, and age (pediatric) have all been shown to increase resuscitation needs in larger burns and may increase needs in smaller burns as well.^10–15^

Despite the lack of evidence-based guidelines for fluid resuscitation in smaller burns, this practice is recommended by the International Society of Burn Injury in their guidelines, but only in the pediatric population.^10^ In one of the only studies comparing lower resuscitation TBSA triggers, 2 of the 5 pediatric burn centers studied, used a resuscitation trigger of 15% compared to the others starting at TBSA ≥20%. Of those 2 centers, one reported the lowest administered resuscitation fluid per body weight and TBSA; the other center’s resuscitation was on par with that of the other 3 centers, all of which had a ≥20% TBSA trigger. The outcomes for patients, especially adults, who undergo intravenous resuscitation for burns under 20% TBSA are otherwise not well studied.

At our institution, patients who sustain burns with TBSA ≥15% undergo an algorithmic fluid resuscitation guided by the PF and titrated based on hourly urine output (UOP). This has been a long-standing practice that was instituted to streamline care at night and to prevent the anecdotal acute kidney injury that occurred in the absence of resuscitation. We sought to expand the literature in small burn resuscitation by reviewing our process and characterizing the outcomes of patients with 15-19.9% burn injuries who underwent fluid resuscitation.

## METHODS

### Ethics statement

This study was approved by our Institutional Review Board. A waiver of consent was approved for all subjects.

### Study design

Our burn registry was queried to identify patients aged ≥18 years old who were admitted between January 1, 2019 and August 29, 2021, with a burn injury affecting between 15 to 19.9% TBSA. Patients were included if they received algorithmic fluid resuscitation via Brooke (BF: 2 mL/kg/TBSA), Parkland (PF), or other formula. Patients were classified as receiving other formula if they underwent algorithmic resuscitation, but starting and titrating volumes were not based on either the PF or BF. Per the algorithm, patients who met UOP targets were progressively weaned to maintenance (40 mL plus body weight in kg, per long-standing unit protocol). Patients who died within 24 h postadmission or had comfort measures instituted were excluded. Patients were also excluded if volume of fluids given prior to reaching maintenance was unavailable in the electronic medical record, for instance, in patients who were given only maintenance fluids, or no fluids were given.

### Study setting: resuscitation protocol

On admission, Lund Browder diagrams were drawn in duplicate, compared, and adjusted for a final %TBSA at admission. The patient was weighed on a hydrotherapy cart. During the study period, we transitioned from BF resuscitation to PF starting at 4 mL/kg/%TBSA of lactated Ringer’s solution (LR) secondary to an audit which showed longer resuscitation times in our larger burn patients with BF.^16^ Fluid rates were adjusted according to the nursing driven protocol every 1 h to maintain UOP between 30 and 50 mL/h. Albumin (5%) was initiated at 20 cc/kg/24 h by protocol or at the discretion of the attending. By protocol, albumin was given for the following criteria: minimal or no UOP after increasing fluids; fluids received by patients since admission were equal to or over half of the estimated 24 h fluids at 8-h mark; persistent acidosis; increasing abdominal or pulmonary pressures and attending discretion; albumin could be started at any time when these parameters were reached (see Appendix 1 for full protocol). Maintenance fluids were continued for 8 h after resuscitation was completed and albumin, if started, was weaned.

The time to reach resuscitation completion was defined as the time the patient reached maintenance fluid rates. Total LR received from admission to time of maintenance was used to calculate resuscitation volumes.

### Data collection

Demographics (age, sex, BMI), comorbidities, injury information (mechanism of burn, %TBSA second and/or third-degree burn), time of injury, time of admission, mode of transportation to the burn center, hospital course (hospital length of stay (LOS), ventilator days, discharge disposition), presence of INH, complications, and mortality were obtained from our institution burn registry. Complications included those anticipated to be associated with resuscitation including cardiac, respiratory, renal, and compartment syndrome. Outcomes included ventilator days, LOS and death. Electronic medical records were reviewed to collect which resuscitation formula was used, predicted 24 h resuscitation goal and final resuscitation fluids received, hourly fluid intake and output, receipt of albumin and/or vasopressor during initial resuscitation, serum lactate and creatinine on admission and at 24 h, and whether the patients underwent surgery for their burn injury.

### Statistical analysis

Parkland formula was used as the reference burn resuscitation formula for all patients. Fluid resuscitation was categorized as below range (<3 mL/kg/%TBSA), within range (3-5 mL/kg/%TBSA or above range (>5 mL/kg/%TBSA). Similarly, UOP was expressed as below range (<30 mL/h), within range (31-50 mL/h) or above range (>50 mL/h). In order to better elucidate how much extra fluids patients received, we correlated the fluid volumes to their predicated maintenance fluid needs as calculated using BSA:

(1500 mL × BSA) + [(25 + %TBSA) × BSA × 24] m^2^ = fluids per 24 h.^17^

Fluid volumes were considered within range for maintenance fluids if the volume fell within 10% of maintenance by ideal body weight. Intake/output (I/O) ratios were calculated using the technique described by Kelly and modified by Lawrence.^18^^,^^19^ The I/O was calculated by dividing 4/weight in kg/%TBSA by UOP (30 or 50 mL)/weight in kg to achieve a unitless variable that varies only with %TBSA. Binary logistic regression including demographics, burn size, ventilator days, and comorbidities was performed to determine independent variables associated with receiving predicted or over-burn formula. Categorical variables were compared using chi-square and Fisher’s exact tests and continuous variables using the Mann–Whitney *U*-test. *P* < .05 was considered significant.

## RESULTS

Of the 43 patients with a burn between 15% and 19.9% TBSA admitted to the burn unit during the study period, one patient died within 24 h postadmission and 9 patients did not receive algorithmic resuscitation. Thirty-three patients received resuscitation fluids and were included in the study. The study population was mostly male (27/33, 81.8%) with a median age of 57 years (ranging from 18 to 88 years). The median burn size was 17%. Most presented with comorbidities (23/33, 69.7%) (Table 1). Six patients (18.2%) required vasopressors and 16 (48.5%) received albumin during their resuscitation. Fifteen patients (45.5%) experienced complications with 4 patients (12.1%) dying.

**Table 1:** Overall Population Description

**Variables**	**Cohort *n* = 33**
Male, *n* (%)	27 (81.8)
Age, median [range]	57 [18-88]
BMI, median [range]	28.6 [16.9-48.4]
Transfer, *n* (%)	29 (87.9)
TBSA, median [range]	17 [15-19]
2nd degree, median [range]	15.5 [0-18.5]
3rd degree, median [range]	1 [0-17]
Inhalation injury, *n* (%)	5 (15.2)
Comorbidities, *n* (%)	23 (69.7)
Diabetes	4 (12.1)
Current smoker	11 (33.3)
Hypertension	13 (39.4)
COPD	1 (3)
Chronic renal failure	0
Atrial fibrillation	0
CHF	0
Alcohol use	2 (6.1)
Substance use disorder	1 (3)

Abbreviations: BMI, body mass index; CHF, congestive heart failure; COPD, chronic pulmonary disease; 2nd, second degree; 3rd, third degree; TBSA, total body surface area.

Of the 33 patients, 15 patients (45%) were considered below the predicated burn formula range, while another 15 (45%) were considered within the burn formula range and the remaining 3 (9%) were considered over burn formula range. As shown in Table 2, patients considered within or over the burn formula range tended to be older (*P* = .077). There were no significant differences between the groups in terms of burn injury severity, presence of INH, or comorbidities. When we looked at those receiving their predicated burn resuscitation or over compared to those who did not, age tended to be higher (38 [27-62] vs 61.5 [37-77.5], *P* = .052). Age remained the only independent variable associated with resuscitation needs, the risk of requiring resuscitation increasing by 3.7% for every year of age (odd ratio = 1.037 [95%CI 1.001-1.074], *P* = .042).

**Table 2:** Outcomes

**Variables**	**Cohort (*n* = 33)**
Complications, *n* (%)	15 (45.5)
Heart complications	13 (39.4)
Cardiac arrhythmia	10 (30.3)
Cardiac ischemia	3 (9.1)
Renal complications	5 (15.2)
ARDS	2 (6.2)
Respiratory failure	8 (24.2)
Compartment syndrome	0
Vent days, median [range]	2.5 [1-16]
Surgery, *n* (%)	23 (69.7)
LOS, median [range]	14 [2-74]
Mortality, *n* (%)	4 (12.1)

Abbreviation: ARDS, acute respiratory distress syndrome; LOS, length of stay.

There was no significant difference in terms of complications or mortality between the groups. Four patients died in the hospital, 4 from cardiac events and sequela that occurred prior to admission and one from early sepsis secondary to a bloodstream infection, but after the resuscitation period. Patients who were under or within the burn formula range had significantly shorter LOS than patients who were over the burn formula (10 vs 17 vs 23 days, *P* = .033, Table 3).

**Table 3:** Comparison of Patients Based on Resuscitation Received

	**Burn formula**			
	**Under**	**Within range**	**Over**	
**Variables**	**(*n* = 15)**	**(*n* = 15)**	**(*n* = 3)**	***P*-value**
Male, *n* (%)	12 (80)	13 (86.7)	2 (66.7)	.693
Age [IQR]	38 [18-83]	60 [24-86]	59 [59-88]	.077
BMI [IQR]	28.6 [16.9-41.8]	30.6 [20.5-48.4]	25.1 [25.1-28.2]	.382
TBSA, median [range]	17 [15-19]	17 [15-19]	17 [17-18]	.705
2nd degree TBSA, median [range]	15.5 [0-18.5]	15 [0.5-18]	5 [5-17]	.603
3rd degree TBSA, median [range]	0 [0-17]	2 [0-16.5]	0 [0-13.5]	.402
Inhalation injury, *n* (%)	1 (6.7)	3 (20)	1 (33.3)	.645
Comorbidities, *n* (%)	11 (73.3)	9 (60)	3 (100)	.356
Diabetes	2 (13.3)	1 (6.7)	1 (33.3)	.426
Current smoker	6 (40)	4 (26.7)	1 (33.3)	.741
Hypertension	4 (26.7)	7 (46.7)	2 (66.7)	.319
COPD	0	1 (6.7)	0	.536
Alcohol use	0	2 (13.3)	0	.279
Substance use disorder	1 (6.7)	0	0	.539
Burn-related surgery, *n* (%)	9 (60)	12 (80)	2 (66.7)	.488

Abbreviations: BMI, body mass index; COPD, chronic obstructive pulmonary disease; TBSA, total burn surface area; 2nd, second degree; 3rd, third degree; No patient had renal failure, atrial fibrillation, or congestive heart failure.

**Figure 1:**
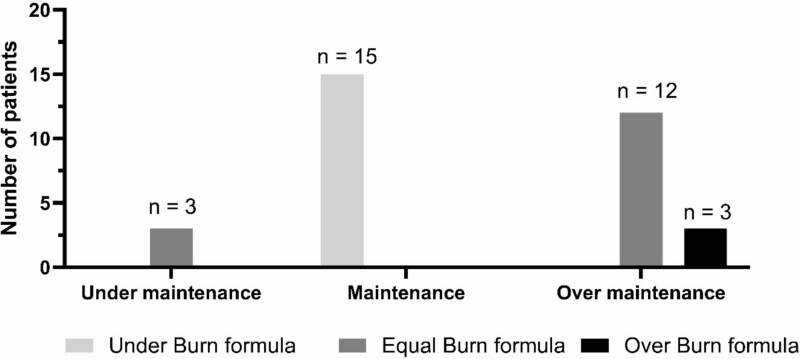
Relationship between Maintenance Fluid and Burn Formula Resuscitation in the Study Population

Comparing the total fluid received to predicted maintenance, just under half of the patients (15, 45%) received maintenance; these patients also received total fluids under their predicted burn formula range. Only 3 patients received total fluids that were under maintenance; all of them were within the burn formula range (3/18). However, the majority of those who were within the burn formula range or over were over maintenance (12/18 [66.7%], Figure 1). Most patients in each group had an average UOP >50 mL/h for the resuscitation period, representing three-fourths of the population (25/33; 75.8%). As shown in Table 3, median lactate and creatinine levels were 2 mmol/L and 0.9 mg/dL on admission, respectively, and remained normal 24 h after admission with no significant difference between the groups (Table 4).

**Table 4:** Patient Outcomes Based on Resuscitation Received

	**Burn formula**			
	**Under**	**Within normal range**	**Over**	
**Variables**	**(*n* = 15)**	**(*n* = 15)**	**(*n* = 3)**	***P*-value**
Clinical laboratory information				
Lactate, initial (mmol/L), median [range]	1.9 [1.5-2.6]	1.8 [1.2-2.8]	1.7 [1.6-.]	.771
Lactate, 24 h (mmol/L), median [range]	1.7 [1.5-.]	2.1 [1.25-2.8]	2.05 [1.5-.]	.801
Lactate max (mmol/L), median [range]	2.8 [1.5-5.6]	3.35 [1.98-4.7]	2.6 [1.6-.]	.645
Creatinine, initial (mg/dL), median [range]	0.9 [0.9-1]	0.9 [0.8-1.1]	1.3 [1.1-.]	.162
Creatinine 24 h (mg/dL), median [range]	0.9 [0.9-1.05]	1 [0.8-1.2]	0.9 [0.9-.]	.143
UOP, median, n (%)				.283
<30 mL/h	1 (6.7)	1 (6.7)	1 (33.3)	
30-50 mL/h	1 (6.7)	4 (26.7)	0	
>50 mL/h	13 (86.7)	10 (66.7)	2 (66.7)	
Complications, n (%)	8 (53.5)	7 (46.7)	1 (33.3)	.907
Heart complications	5 (33.3)	7 (46.7)	1 (33.3)	.737
Cardiac arrhythmia	3 (20)	6 (40)	1 (33.3)	.488
Cardiac ischemia	1 (6.7)	2 (13.3)	0	.693
Renal complications	2 (13.3)	2 (13.3)	1 (33.3)	.654
ARDS	1 (6.7)	0	1 (33.3)	.086
Respiratory failure	3 (20)	4 (28.7)	1 (33.3)	.848
Compartment syndrome	0	0	0	
Vasopressor, *n* (%)	1 (6.7)	4 (26.7)	1 (33.3)	.283
Albumin, *n* (%)	4 (26.7)	9 (60)	3 (100)	.033
LOS (days), median [range]	10 [2-21]	17 [3-74]	23 [3-24]	.033
Ventilator days, median [range]	2 [1-4]	2 [1-16]	3 [3-3]	.635
Burn surgery, *n* (%)	9 (60)	12 (80)	2 (66.7)	.488
Mortality, *n* (%)	1 (6.7)	2 (13.3)	1 (33.3)	.426

Abbreviations: ARDS, acute respiratory distress syndrome; BSA, body surface area; LOS, length of hospital stay; UOP, urine output.

We further characterized the resuscitations of the 12 patients who were within their predicted burn formula range and the 3 who were over, all of whom were over maintenance (Figure 1).

**Figure 2:**
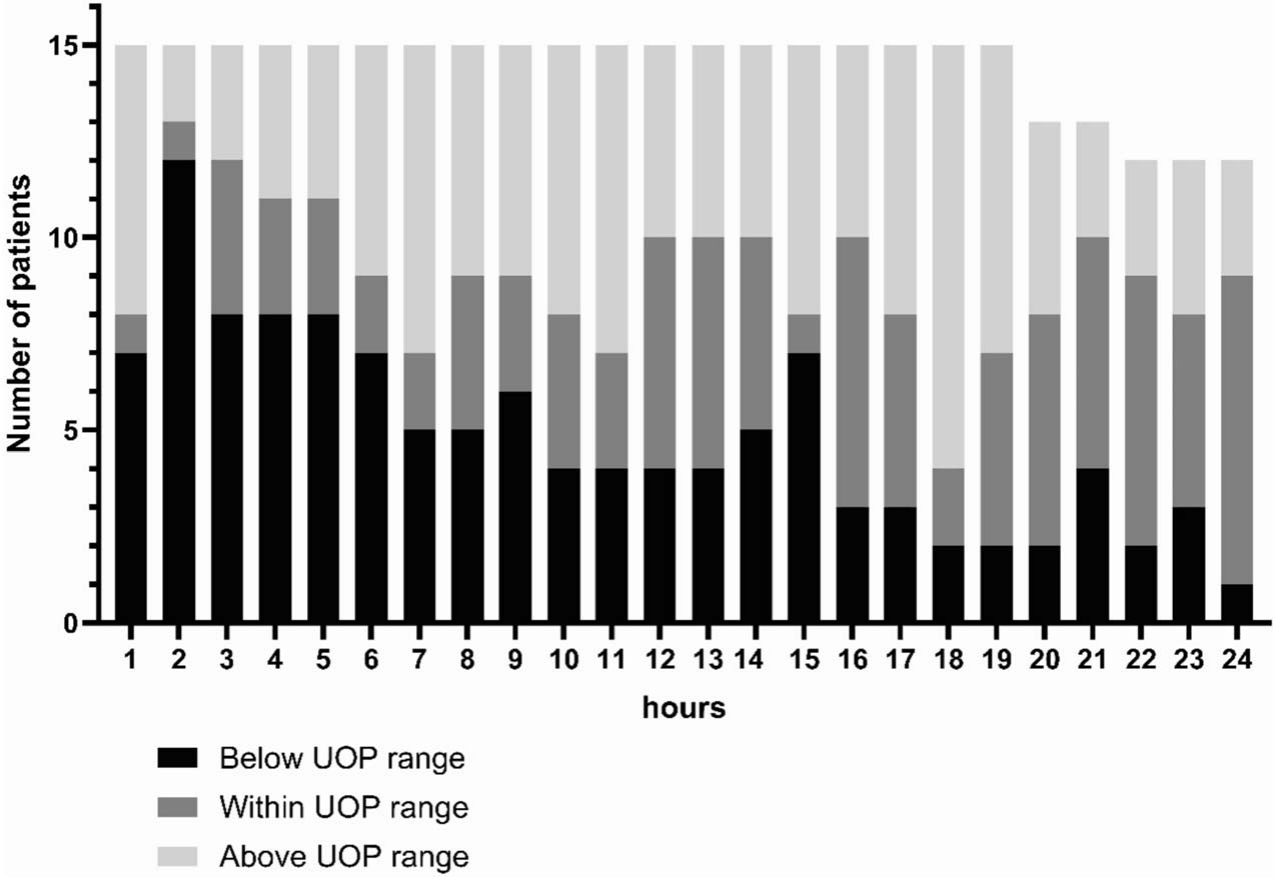
Average UOP for Patients Who Were Within or Over-Burn Formula Resuscitation and Over Maintenance during the Resuscitation Period (15 Patients)

**Figure 3:**
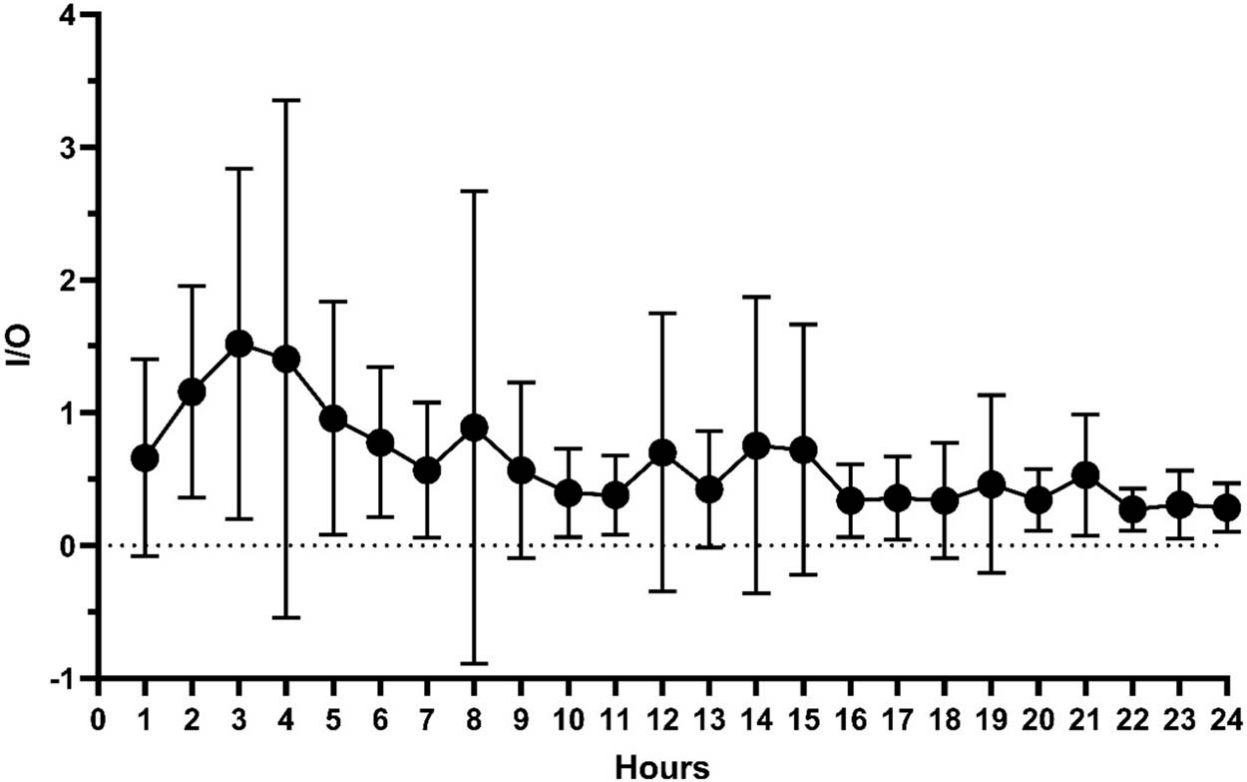
Average I/O Ratios of Patients Who Were Within or Over-Burn Formula Resuscitation and Over Maintenance during the Resuscitation Period (15 Patients)

**Figure 4:**
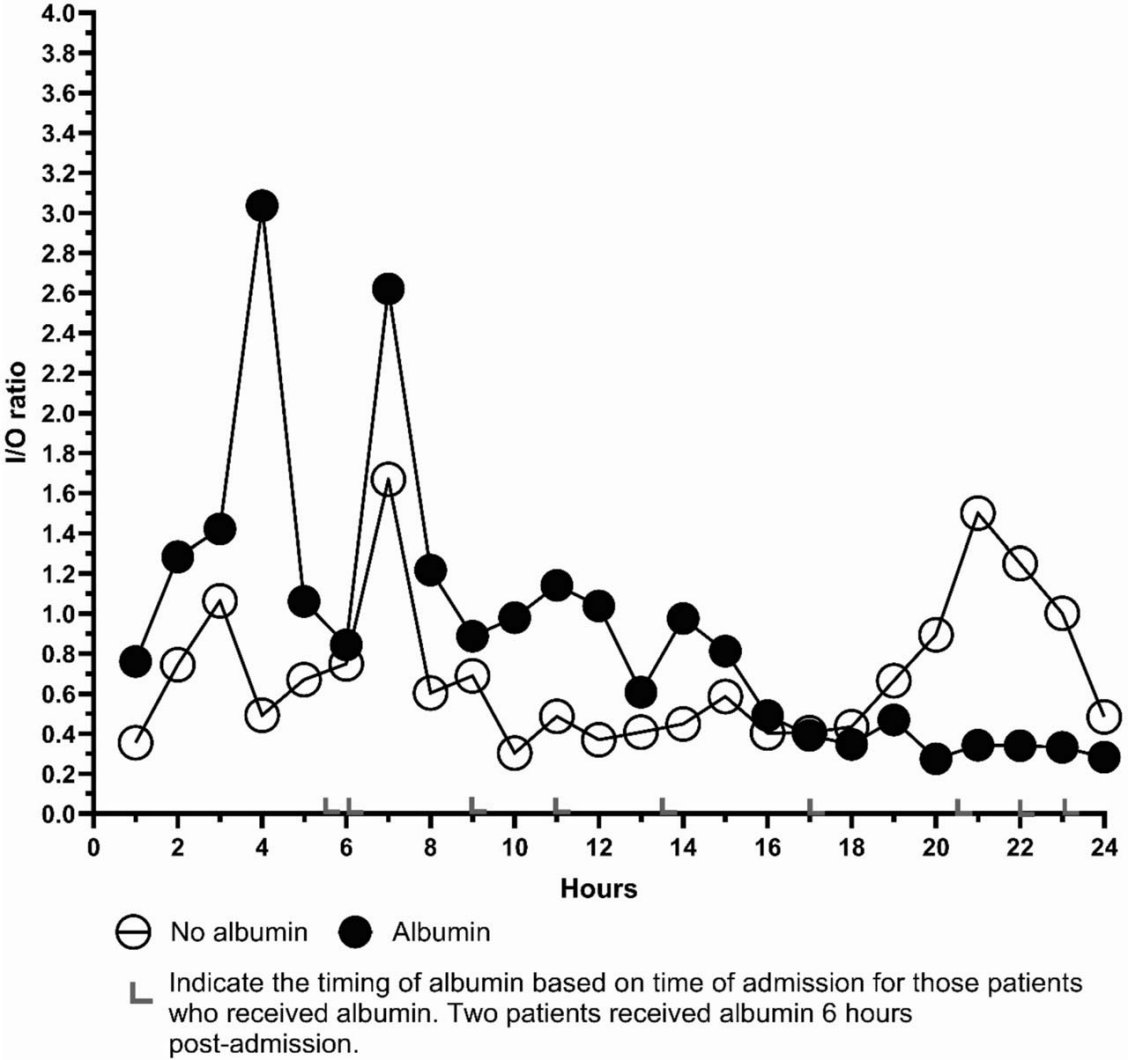
Comparison of I/O Ratio of Patients Who Did Not Receive Albumin (Open Circles) Compared to Those Who Did Receive Albumin (Closed Circles) Who Were Within or Over-Burn Formula Resuscitation and Over Maintenance

The UOP in this group remained variable but showed a normalizing trend as the resuscitation period progressed (Figure 2). To better elucidate the resuscitation given we evaluated hourly I/O ratio in this group. As shown in Figure 3, average I/O ratios varied between patients and during the first 24 h. The median I/O ratio for the cohort was 0.056 which was considerably lower than that estimated by the Lawrence method yielding 2.22 for a UOP of 30 mL/h or 1.36 for a UOP of 50 mL/h. Two-thirds of this group received albumin at different time points during the first 24 h postadmission (Figure 4).

## DISCUSSION

Resuscitation remains an ongoing topic of discussion and investigation in the management of burned patients.^3^^,^^7^ Centers are consistently attempting to fine tune their goal directed protocols to provide patients with sufficient fluid to avoid burn shock, while reducing overall fluid administration.^20^^,^^21^ While the ABRUPT trial provided insights into the practices of multiple centers regarding crystalloid and colloid resuscitation, the focus was entirely on patients with burn size of 20% and greater.^22^ To date, there is limited study of resuscitation of burns <20% and none in adults. In this study, we reviewed our practice of resuscitation of burns starting at 15% TBSA. We found that just under half of these patients (45%) received fluid as predicted by burn formula with a small number receiving greater than burn formula-predicted volumes (10%). Moreover, all but 3 patients in the current study received fluid volumes that were equal to or over their predicted burn-based maintenance formula. This is a population currently missed by the American Burn Life Support recommended resuscitation trigger of >20% TBSA or greater. Herein, we sought to describe this population.

Giving the correct amount of fluid is paramount to successful outcomes of burn patients. The provision of excess resuscitation fluids has been an ongoing problem regardless of the resuscitation formula used, albumin administered, or adjuncts given. Over-resuscitation is not without consequences and can lead to serious morbidity including death.^23^ Almost one-half of the patients in the current study received their predicted burn resuscitation and 10% received more fluids than predicted. On multivariable analysis, only age was associated with the need for resuscitation, increasing the need by 3.7% for each year. This group also required vasopressors more often than the group that did not require resuscitation. Yet, there was no difference in complications between the groups based on resuscitation fluid received. While it is hard to definitively report the success or the harm of resuscitation in this population given the retrospective design and small cohort, the 24-h normal median creatinine and lactate levels in the patients’ who received their predicted resuscitation volumes may support the necessity of the fluid given in this critically ill patient population.

Another finding of our study was the surprising number of patients who required albumin. Patients receiving fluids that were either within range or over the burn resuscitation formula received significantly more albumin than the group who received under the burn formula predictions, with two-thirds (66.7%) of the latter group (12 of 18 patients) receiving albumin during their resuscitation compared to 26.7% in the under-burn resuscitation group (4/15). Similar to the receipt of resuscitation of predicted volumes, the receipt of albumin tended to be related to age (*P* = .063). Albumin has previously been shown to normalize the I:O ratio of run-away resuscitations in multiple studies in burns >20% TBSA or greater.^19^^,^^24^^,^^25^ The ABRUPT study demonstrated that patients who were more likely to require colloid fluid were older and had more comorbidities.^22^ This study, as well as others, informed the American Burn Association Clinical Practice Guidelines to recommend albumin use as a rescue where resuscitation is deteriorating despite receiving escalating amounts of cyrstalloids.^7^ The recommendation was stronger for patients with large burns. In our protocol, albumin is used as a rescue fluid for those who meet preset criteria. (Appendix 1) It is important to recognize that the median age of our population was 57 years old which is considerably older than the national mean age 41 years.^26^ Given the small sample size, it is hard to draw any conclusions regarding why the latter 2 groups either received burn formula-predicted volumes or over-prediction volumes predicted and received more albumin supplementation.

Our study is limited by several factors. It is a single-center study with an overall small number of patients included. Additionally, we are limited by the retrospective chart review nature of the study and the inability to adequately capture the clinical reasons for ongoing resuscitation. The study also reviewed a period where we shifted from BF to PF; this could have confounded our results. Moreover, 30% were resuscitated by initial volumes that did not match either formula. However, the use of a specific burn formula does not appear to result in a significant decrease in the fluids ultimately received.^22^^,^^27^ Additionally, we are limited to the documentation in the charts regarding comorbidities and complications and are unable to render a judgment regarding severity. Due to the size and design of the study, we cannot conclude that resuscitation aided or harmed our patients and simply present our observations. However, based on these limited observations in a population that is considerably older than the average burn population with a high rate of comorbidities, resuscitation does not appear to impose harm in our small study cohort.^26^

## CONCLUSION

In conclusion, in our review of patients who received resuscitation for burn sizes 15%-19.9%, we show fidelity to our protocol with most patients receiving their predicted volume or less, no difference in complications between the 3 groups based on the fluid volume they received (under, within, or over the predicted burn formula resuscitation volume) and that most patients received more than their burn-based maintenance or resuscitation. This study, with a median age of 57 years which is older than the national mean burn age of 41, suggests the need for more focus on elderly burns and perhaps a lower resuscitation trigger like that proposed for pediatric burn patients.^10^ The answer to who needs resuscitation and how to give it for burns <20% needs to be definitively answered in a larger, prospective study.

## Supplementary Material

Flowsheet_Combined_iraf185

## Notes

Author contributions: Ashleigh Bull (Conceptualization, Data curation, Formal analysis, Writing—original draft, Writing—review & editing: [equal]), Mala Sharma (Conceptualization [supporting], Data curation [equal], Formal analysis [supporting], Writing—original draft, Writing—review & editing [equal]), Alexander Kurjatko (Conceptualization, Data curation [equal], Formal analysis [supporting], Writing—original draft, Writing—review & editing [equal]), Sarah Wellsandt (Conceptualization [equal], Formal analysis, Writing—original draft [supporting], Writing—review & editing [equal]), Brooke Dwars (Conceptualization [equal], Writing—original draft [supporting], Writing—review & editing [equal]), Colette Galet (Conceptualization [equal], Data curation[supporting], Formal analysis, Methodology [lead], Writing—original draft, Writing—review & editing [equal]), and Lucy Wibbenmeyer (Conceptualization [lead], Data curation [supporting], Project administration [lead], Writing—original draft [equal], Writing—review & editing [equal])

Funding: None declared.
