# Supplementary material for: Small Burns Need Attention Too: Evaluating the 15% Burn Resuscitation Threshold in Adults
Source: J Burn Care Res. 2025 Sep 29;47(1):403–9. doi: 10.1093/jbcr/iraf185 (PMC12770978; doi:10.1093/jbcr/iraf185)
Supplement: Flowsheet_Combined_iraf185 [file flowsheet_combined_iraf185.pdf]

ALL Burn Patients (>30 Kg)
Protocol for Fluid Resuscitation

Fluid Resuscitation Formula for Patients with TBSA ≥15%:

Age:	Parkland Formula:
Adults and pediatrics > 30kg	4ml/kg/%BSAB

*Do not include albumin, tube feedings, D5LR, or Meds as part of the resuscitation fluids- you should only titrate the LR during the resuscitation phase

Calculate the IVY Index (250ml/kg):

250ml x _______= _____________
(weight-kg)

Admit weight:_______.___kg
Burn size: __________.___%

Parkland Formula for Fluid Resuscitation

______ kg x _______ x 4 = ____________
(weight) (BSAB)
________ mls / 1st 24 hours
/2 = ________ mls / 1st 8 hours
/8 = ________ mls / hr IV rate

Starting fluid rate:_____**mL**

Maintenance IVF Rate:
Wt (kg) + 40 = _____ml/hr

All Burn Patients with **BSAB ≥ 20%** need **DHT** on Admission. Goal is to start feeds **within 6 hours** of admission

All Burn Patients with a **BSAB ≥ 30%** need **Bladder Pressures** checked **q2 hours**

Step One:
Infuse LR at calculated rate per Brooke formula (below)
Measure Urine Output hourly

→ Unstable Vital Signs:
HR > 140, < 60
BP < 90/60
SaO2 < 90;
CALL PHYSICIAN

Vital Signs Stable: HR < 140, BP > 90/60, SaO2 > 90%

- Urine Output <15 mL → Increase IV rate by 20% initial rate +_______ml → If urine output < 15 mL/hr for 2 consecutive hours, critical edema, low BP, or has failure of resuscitation signs listed below: Obtain ABG, Lactate, Bladder Pressure **CALL BURN ATTENDING**
- Urine Output 16-30 mL → Increase IV rate by 10% initial rate +_______ml
- Urine Output 31-50 mL → No change in IV rate
- Urine Output 51-100 mL → Decrease IV rate by 10% initial rate -_______ml
- Urine Output >101 mL → Decrease IV rate by 20% initial rate -_______ml → Urine output > 101 mL/hour for 2 consecutive hours: **CALL BURN ATTENDING**

Failure of Resuscitation Signs- Consider Albumin Protocol:

Minimal or no UOP after increasing Fluids
I>O
Fluids received by patient since admission are equal to over half of the estimated 24 hours fluids at the 8-hour mark
Persistent Acidosis
Increasing abdominal or pulmonary pressures

Albumin Protocol
Adults: Run 5% Albumin at 20 ml/kg/24 hours
20 x _______kg = __________
/24 = ___________
Albumin rate = __________ml/h
** this is run continuously, not as a bolus
** Once LR is at maintenance, communicate with Burn Attending about weaning albumin off

Repeat **Step One** every hour until maintenance rate is reached:
Calculated Maintenance Rate = 40 mL + dry weight (kg) =_________total mL/hour

IF IV fluid rate is 50% above starting fluid rate: START ALBUMIN PROTOCOL
0.5 x ________ = _______ +_________= ________ml is **50% Above Starting Rate**
(Starting rate) (starting rate)

Keep at Maintenance IV fluid rate for 8 hours. Continue hourly UOP during this period.

After 8 hours at maintenance rate, wean IV fluid down by 20ml q2 hours as long as PO/Feedings intake supports adequate UOP. Monitor UOP q1 hours until IV fluids are off.

If urine output falls below 30 mL/hr return to **Step One** & **CALL PHYSICIAN**
Obtain ABG, lactate, and Bladder Pressure

DATE: ______________________

Burn Treatment Center - 8JCP
Hourly Flowsheet

Bar Code Label

Place in ANM mailbox

Total Parkland 24H:

	Intake Values										Output		Lab Values						
Time	Total LR Rate	IV # 1 LR	IV # 2 LR	IV # 3 LR	IV # 4 Albumin	IV # 5 D5LR per peds protocol	IV # 6 Sedation	IV # 7 Pain	DHT Feeds	LR Med Vol Q 1	Urine Output	NG Out	CVP	Bladder Pressure	ABG	Lactate	pH	Blood Glucose	PRNs/ Other
2300																			
2400																			
0100																			
0200																			
At 0200: # of Hours from Start of Resuscitation:										LR Total from Start of Resuscitation:						%Parkland=			
0300																			
0400																			
0500																			
0600																			
At 0600: # of Hours from Start of Resuscitation:										LR Total from Start of Resuscitation:						% Parkland=			
0700																			
0800																			
0900																			
1000																			
At 1000: # of Hours from Start of Resuscitation:										LR Total from Start of Resuscitation:						% Parkland=			
1100																			
1200																			
1300																			
1400																			
At 1400: # of Hours from Start of Resuscitation:										LR Total from Start of Resuscitation:						% Parkland=			
1500																			
1600																			
1700																			
1800																			
At 1800: # of Hours from Start of Resuscitation:										LR Total from Start of Resuscitation:						% Parkland=			
1900																			
2000																			
2100																			
2200																			
At 2200: # of Hours from Start of Resuscitation:										LR Total from Start of Resuscitation:						% Parkland=			
